# Depression was associated with younger age, female sex, obesity, smoking, and physical inactivity, in 1027 patients with newly diagnosed type 2 diabetes: a Swedish multicentre cross-sectional study

**DOI:** 10.1186/s12902-022-01184-3

**Published:** 2022-11-09

**Authors:** Eva O. Melin, Pär Wanby, Thomas Neumark, Sara Holmberg, Ann-Sofi Nilsson Neumark, Karin Johansson, Mona Landin-Olsson, Hans Thulesius, Magnus Hillman, Maria Thunander

**Affiliations:** 1 Department of Clinical Sciences, Diabetology and Endocrinology, Lund University, Lund, Sweden; 2 Diabetes Research Laboratory, Biomedical Centre, Lund University, Lund, Sweden; 3 Department of Research and Development, Region Kronoberg, Box 1223, 351 12 Växjö, Sweden; 4 Region Kronoberg, Primary Care, Växjö, Sweden; 5 Department of Medicine and Optometry, Linnaeus University, Kalmar, Sweden; 6 Department of Medical and Health Sciences, University of Linköping, Linköping, Sweden; 7 Department of Internal Medicine, Endocrinology, Kalmar County Hospital, Region Kalmar, Sweden; 8 Regional Executive Office - Coordination of Health Care, Kalmar, Sweden; 9 Department of Research, Region Kalmar County, Kalmar, Sweden; 10 Department of Laboratory Medicine, Division of Occupational and Environmental Medicine, Lund University, Lund, Sweden; 11 Primary Care, Mönsterås, Sweden; 12 Department of Health and Caring Sciences, Linnaeus University, Växjö, Sweden; 13 Department of Diabetology and Endocrinology, Skane University Hospital, Lund, Sweden; 14 Department of Clinical Sciences, Division of Family Medicine, Lund University, Malmö, Sweden; 15 Department of Internal Medicine, Endocrinology and Diabetes, Växjö Central Hospital, Region Kronoberg, Växjö, Sweden

**Keywords:** Age, Antidepressants, Anxiety, Depression, Epidemiology, Obesity, Physical inactivity, Sex differences, Smoking, Type 2 diabetes

## Abstract

**Background:**

Depression is a risk factor for type 2 diabetes (T2D) and cardiovascular disease (CVD). The aims were to explore the prevalence of depression, anxiety, antidepressant use, obesity, Hemoglobin A1c > 64 mmol/mol, life-style factors, pre-existing CVD, in patients with newly diagnosed T2D; to explore associations with depression; and to compare with Swedish general population data.

**Methods:**

Multicentre, cross-sectional study. Inclusion criteria: adults with serologically verified newly diagnosed T2D. Included variables: age, sex, current depression and anxiety (Hospital Anxiety and Depression Scale), previous depression, antidepressant use, obesity (BMI ≥ 30 and ≥ 40 kg/m^2^), Hemoglobin A1c, pre-existing CVD. Logistic regression analyses were performed.

**Results:**

In 1027 T2D patients, aged 18–94 years, depression was associated with age (per year) (inversely) (odds ratio (OR) 0.97), anxiety (OR 12.2), previous depression (OR 7.1), antidepressant use (OR 4.2), BMI ≥ 30 kg/m^2^ (OR 1.7), BMI ≥ 40 kg/m^2^ (OR 2.3), smoking (OR 1.9), physical inactivity (OR 1.8), and women (OR 1.6) (all *p* ≤ 0.013).

Younger women (*n* = 113), ≤ 59 years, compared to younger men (*n* = 217) had higher prevalence of current depression (31% *vs* 12%), previous depression (43 *vs* 19%), anxiety (42% *vs* 25%), antidepressant use (37% *vs* 12%), BMI ≥ 30 kg/m^2^ (73% *vs* 60%) and BMI ≥ 40 kg/m^2^) (18% *vs* 9%), and smoking (26% *vs* 16%) (all *p* ≤ 0.029).

Older women (*n* = 297), ≥ 60 years, compared to older men (*n* = 400) had higher prevalence of previous depression (45% *vs* 12%), anxiety (18% *vs* 10%), antidepressant use (20% *vs* 8%), BMI ≥ 30 kg/m^2^ (55% *vs* 47%), BMI ≥ 40 kg/m^2^ (7% *vs* 3%) (all *p* ≤ 0.048), but not of current depression (both 9%).

Compared to the Swedish general population (depression (women 11.2%, men 12.3%) and antidepressant use (women 9.8%, men 5.3%)), the younger women had higher prevalence of current depression, and all patients had higher prevalence of antidepressant use.

**Conclusions:**

In patients with newly diagnosed T2D, the younger women had the highest prevalence of depression, anxiety, and obesity. The prevalence of depression in young women and antidepressant use in all patients were higher than in the Swedish general population. Three risk factors for CVD, obesity, smoking, and physical inactivity, were associated with depression.

## Background

The comorbidity of depression and diabetes mellitus is associated with increased cardiovascular disease (CVD) and all-cause mortality [1]. A bidirectional link between depression and type 2 diabetes (T2D) has been demonstrated, but depression seems to be a stronger risk factor for T2D than the other way round [2, 3]. The presence of anxiety in depressed people seems to further increase the risk of developing T2D [4]. Depression is, however, a heterogenous disorder characterized by dysphoria, anhedonia and/or lack of interest, accompanied by cognitive symptoms, increased or decreased appetite, weight gain or weight loss, hypersomnia or insomnia, psychomotor retardation or activation, and fatigue, leading to functional deterioration [5]. Atypical depression, which is more common in women than in men, is characterized by increased appetite, weight gain, fatigue, and hypersomnia, subsequently increasing the risk for obesity with secondary metabolic disturbances [6, 7]. In a Swedish general population survey with 7618 respondents from a random sample of 16 000 people, the prevalence of self-reported depression was higher in men (12.3%) than in women (11.2%) according to the Hospital Anxiety and Depression Scale (HADS), and the prevalence of antidepressant use was higher in women (9.8%/) than in men (5.3%) [8].

T2D is characterized by hyperglycemia caused by resistance to insulin action and an inadequate compensatory insulin secretory response [9, 10]. The incidence of T2D was 399/100 000 inhabitants in Sweden in 2013 [11], and the prevalence of T2D was 6.8% [12]. The onset of T2D is typically slow with a long pre-detection period of 3–7 years [10]. The risk of newly diagnosed T2D increases with age [9], but younger-onset T2D is particularly harmful with increased mortality [13]. Obesity [9, 10, 14], physical inactivity [9, 10], and smoking [15] are other risk factors for incident T2D. The prevalence of depression in patients with newly diagnosed T2D in Sweden has to our knowledge not been previously explored. We hypothesized that the prevalence of depression was high in patients with newly diagnosed T2D, and that depression may be associated with age, sex, obesity, Hemoglobin A1c (HbA1c), life style factors, and/or pre-existing CVD. Our aims were first to explore the prevalence of current and previous depression, anxiety, antidepressant use, obesity, high HbA1c (> 64 mmol/mol), smoking, physical inactivity, pre-existing myocardial infarction (MI), and stroke, while exploring sex and age-related differences in adult patients with newly diagnosed T2D. Second, we explored associations between current depression and all included variables in the T2D patients. Third, we performed comparisons between the T2D patients and Swedish general population data regarding the prevalence of depression, antidepressant use, obesity and smoking.

## Methods

### Participants and study design

**Fig. 1:**
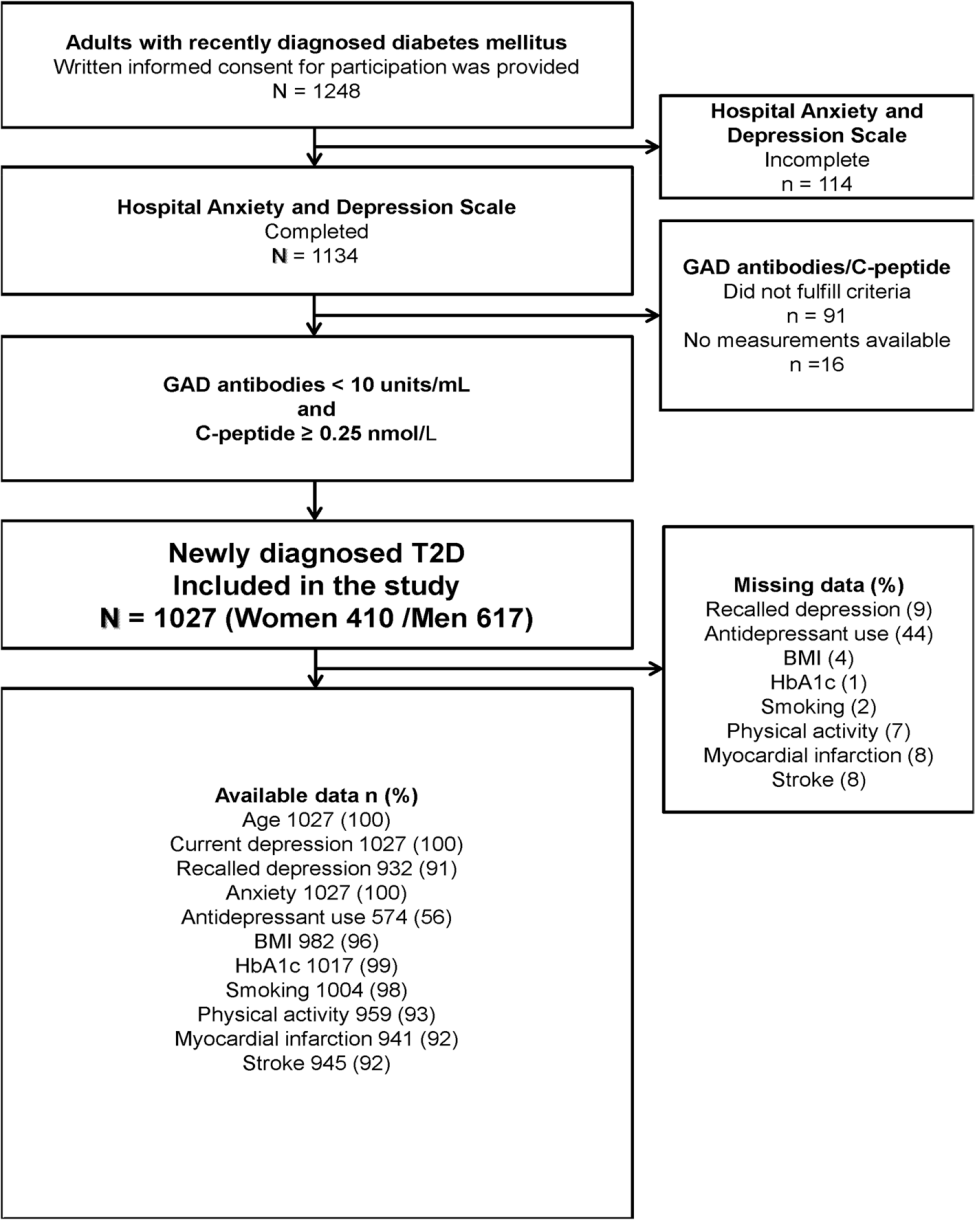
Flow chart for 1027 included patients complemented with presentation of included variables and missing data

Multicentre, cross-sectional design. Inclusion criteria were adults (≥ 18 years) with newly diagnosed serologically verified T2D, and completion of the Swedish version of HADS (see Fig. 1). Exclusion criteria were confirmed diagnosis of type 1 diabetes (T1D) and gestational diabetes. The participants were recruited from all 5 hospitals and 54 primary health care units in Region Kronoberg (193 000 inhabitants) and Region Kalmar (240 000 inhabitants) in South Eastern Sweden. The primary care units served both urban and rural areas. The recruitment period ranged from 1^st^ January 2016 until 31^st^ December 2017 in Region Kronoberg, and from 1^st^ March 2016 until 28^th^ February 2018 in Region Kalmar. The instructions to the health care units were that all patients with newly diagnosed T2D should be informed and offered participation in the study by their physician or diabetes nurse when they received their T2D diagnoses, which could either be performed directly at the health care unit, or later by a telephone call or a letter. If that had not been completed, they should be offered participation at the follow-up visit which routinely takes place within 2–3 weeks after being diagnosed. Out of the 1248 patients who provided consent for participation, 114 patients were excluded due to incomplete HADS testing, and 107 patients were excluded as their clinical diagnoses were not serologically verified (Fig. 1).

Finally, 1027 participants were included constituting 29% of the 3592 patients who were diagnosed with clinical T2D in the two regions during the recruitment periods, 429 out of 1288 (33%) in Region Kronoberg and 595 out of 2304 (26%) in Region Kalmar. Interviews, anthropometrics, biochemical analyses, and data collection from electronic health records (EHRs) were performed.

### Newly diagnosed type 2 diabetes and serological confirmation

The diagnostic criteria for diabetes mellitus were either fasting plasma glucose ≥ 7.0 mmol/L twice within two weeks, a 2-h 75 g post oral glucose tolerance test (OGTT) ≥ 11.1 mmol/L, a random venous glucose ≥ 11.1 mmol/L, or capillary glucose ≥ 12.2 mmol/L in a patient with symptoms of hyperglycemia, or Hemoglobin A1c **(**HbA1c) ≥ 48 mmol/mol (≥ 6.5%) [9, 10].

Newly diagnosed and serologically confirmed T2D was defined as fulfilling the diagnostic criteria for diabetes mellitus without a previous history of a diabetes diagnosis or treatment, with serological confirmation by glutamic acid decarboxylase (GAD) antibodies < 10 units/mL and C-peptide levels ≥ 0.25 nmol/L [16]. GAD antibodies were analysed by using enzyme linked immunosorbent assay (ELISA) from RSR® (Article nr Rs-GDE/96, RSR Ltd, Cardiff, UK) and C-peptide was analysed by commercial ELISA (Mercodia® (article nr 10–1136-01), Uppsala, Sweden), at the Diabetes Laboratory, Lund University, Lund, Sweden, for the purpose of this study.

### High Hemoglobin A1c

HbA1c analyses were performed routinely at the time for the diagnoses by Olympus automated clinical chemistry analysers with high specificity (Olympus AU®, Tokyo, Japan). The HbA1c values were collected from EHRs (Cambio Cosmic®), which were used by all the hospitals and primary care units in the two regions. The intra-coefficient of variation was < 1.2%. High HbA1c was defined as > 64 mmol/mol (IFCC) (> 8% NGSP), the cut-off level corresponded to the 75^th^ percentile.

### Current and previous depression, anxiety and the use of antidepressants

Current depression was defined as HADS-D (the depression subscale) ≥ 8 points, and anxiety as HADS-A (the anxiety subscale) ≥ 8 points [8, 17–20].

The participants were asked whether they had been depressed previously, and whether they used antidepressants. In both cases there were two response options, yes or no. Previous depression was defined as answering yes to the first of these two questions.

### Age

Age was used either as a continuous variable; a dichotomous variable (younger participants (< 60 years) and older participants (≥ 60 years)); or divided into 7 age-groups (18–29, 30–39, 40–49, 50–59, 60–69, 70–79, and 80–94 years).

### Anthropometrics

Weight and length were measured by a nurse according to standard procedures. Obesity was defined as Body Mass Index (BMI) ≥ 30 kg/m^2^ [21], and severe obesity as BMI ≥ 40 kg/m^2^ [22].

### Smoking and physical activity

The patients reported smoking habits as never, previous, non-daily, or daily smokers, which were dichotomized into current smokers (daily and non-daily smokers), and non-smokers (never and previous smokers).

Physical activity was reported as ≥ 30 min of moderate activities performed never, less than once a week, 1–2 times/week, 3–5 times/week, or daily, corresponding to the registration in the Swedish National Diabetes Register (S-NDR) [23]. The levels of physical activity were dichotomized into physical inactivity (less than once a week) and physical activity (all other levels). Both leisure time physical activity and work-related physical activity were taken into account.

### Cardiovascular disease

Patients were interviewed about previous MI and stroke. Data was complemented from the EHRs.

### Total number of newly diagnosed T2D patients in the two regions

The total number of patients with newly diagnosed clinical T2D was collected from the EHRs in Region Kronoberg and Region Kalmar during the recruitment periods. The clinical T2D diagnoses were not systematically serologically confirmed.

### Statistical analysis

As age was not normally distributed, the analyses were performed with Mann–Whitney *U* test, and the results were presented as median (quartile (q)_1_, q_3_). Pearson´s Chi-Squared test, Linear-by- Linear Association, or Fisher´s Exact Test (all two-tailed), were used to analyse categorical data which were presented as *n* (%). Odds ratios (OR) were calculated using logistic regression analyses (simple) with current depression as dependent variable. 95% confidence intervals (CI) were used. *P* < 0.05 was considered statistically significant. SPSS® version 27 (IBM, Chicago, Il, USA) was used.

## Results

In this study of depression in patients with newly diagnosed T2D, 1027 patients aged 18 to 94 years were included (women 40%, born in Sweden 88%). C-peptide levels ranged from 0.25 to 5.58 nmol/L. All patients were GAD antibody negative.

**Fig. 2:**
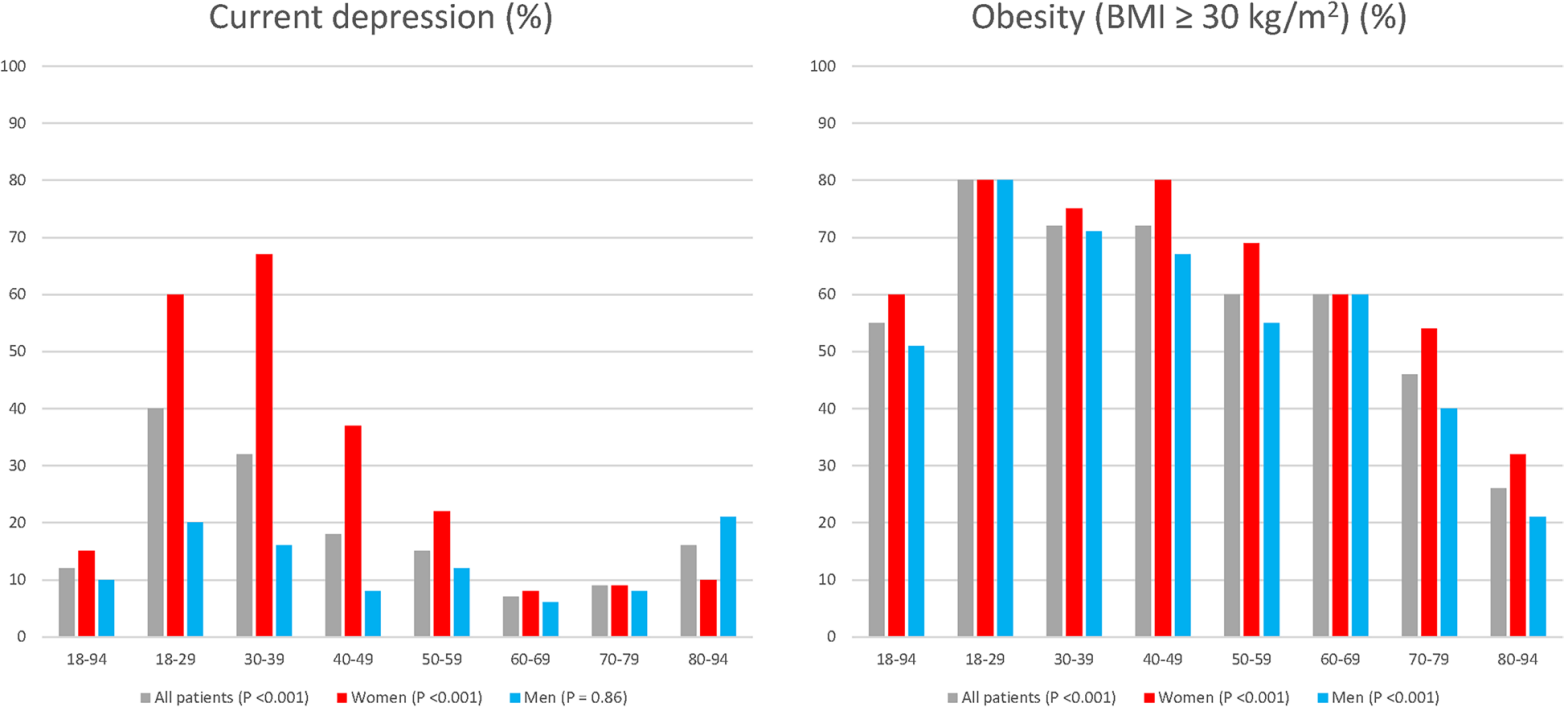
The prevalence of depression and obesity presented gender specified for all and 7 age-groups

The total prevalence of current depression and obesity (BMI ≥ 30 kg/m^2^) and their distribution within seven age-groups are presented for all and for each sex in Fig. 2.

The prevalence of current depression was for all 1027 participants 12%, for 410 women 15%, and for 617 men 10%. The highest prevalence of current depression was found in the two age-groups 18—29 years (all patients 40%, women 60%, men 20%), and declined successively until the age-group 60–69 years (all patients 8%) (*p* < 0.001). The prevalence of obesity was for all patients 55%, for women 60%, and for men 51%. The highest prevalence of obesity was found in the age-group 18—29 years (all patients 80%), and declined successively until the age-group ≥ 80 years (all patients 26%) (*p* < 0.001).

In Table 1, baseline characteristics are presented for all participants, and age and sex specified.

**Table 1:** Presentation of baseline characteristics, including age and sex related differences, for 1027 patients with type 2 diabetes

		**All patients**	**Age-related differences**			**Sex differences**					
		**(18–94 years)**	**Younger** **(18–59 years)**	**Older** **(60–94 years)**		**Younger (18–59 years)**			**Older (60–94 years)**		
					***P***^*** a***^	**Women**	**Men**	***P***^*** a***^	**Women**	**Men**	***P***^*** a***^
*n*		1027	330	697		113	217		297	400	
Sex	Women	410 (40)	113 (34)	297 (43)	0.011	-	-	-	-	-	-
	Men	617 (60)	217 (66)	400 (57)		-	-	-	-	-	-
Age		-	-	-	-	52 (45, 56)	53 (45, 57)	0.37 ^b^	69 (65, 74)	70 (66, 76)	0.065 ^b^
Depression (current) (HADS-D ≥ 8)^ c^		121 (12)	60 (18)	61 (9)	< 0.001	35 (31)	25 (12)	< 0.001	26 (9)	35 (9)	> 0.99
Depression (previous) ^d^		191 (20)	83 (28)	108 (17)	< 0.001	45 (43)	38 (19)	< 0.001	63 (45)	45 (12)	< 0.001
Anxiety (HADS-A ≥ 8)^ e^		195 (19)	102 (31)	93 (13)	< 0.001	48 (42)	54 (25)	0.001	52 (18)	41 (10)	0.005
Antidepressants ^f^		92 (16)	43 (22)	49 (13)	0.003	29 (37)	14 (12)	< 0.001	33 (20)	16 (8)	< 0.001
Obesity (BMI ≥ 30 kg/m^2^) ^g^		538 (55)	203 (65)	335 (50)	< 0.001	81 (73)	122 (60)	0.023	153 (55)	182 (47)	0.048
Obesity (severe) (BMI ≥ 40 kg/m^2^) ^g^		67 (7)	38 (12)	29 (4)	< 0.001	20 (18)	18 (9)	0.017	19 (7)	10 (3)	0.008
HbA1c > 64 mmol/mol (> 8%) ^h^		253 (25)	122 (37)	131 (19)	< 0.001	21 (19)	101 (46)	< 0.001	46 (16)	84 (21)	0.081
Smoking ^i^		127 (13)	61 (19)	66 (10)	< 0.001	28 (26)	33 (16)	0.029	30 (10)	36 (9)	0.64
Physical inactivity ^j^		294 (31)	115 (37)	179 (28)	0.003	36 (33)	79 (39)	0.32	78 (28)	101 (27)	0.78
Myocardial infarction (previous) ^k^		95 (10)	13 (4)	82 (13)	< 0.001	4 (9)	9 (5)	> 0.99 ^l^	19 (7)	63 (17)	< 0.001
Stroke (previous)^m^		62 (7)	8 (3)	54 (8)	0.001^ l^	5 (5)	3 (2)	0.13 ^l^	15 (6)	39 (10)	0.025

Data are presented as *n* (%) or median (q_1_, q_3_)

^a^ Pearson Chi-Square unless indicated. ^b^ Mann–Whitney *U* test. ^c^ Hospital Anxiety and Depression Scale-Depression subscale. Missing values (all/ < 60 years/ ≥ 60 years) (*n*): ^d^ 95/28/67). ^e^ Hospital Anxiety and Depression Scale-Anxiety subscale

Missing values (all/ < 60 years/ ≥ 60 years) (*n*): ^f ^453/138/315. ^g^ Body Mass Index. Missing values (all/ < 60 years/ ≥ 60 years) (*n*): ^g^ 45/16/29. ^h^ Hemoglobin A1c. Missing values (all/ < 60 years/ ≥ 60 years) (*n*): ^h ^10/2/8; ^i^ 23/9/14; ^j ^68/20/48; ^k ^86/28/58; ^l ^Fisher´s Exact Test. Missing values (all/ < 60 years/ ≥ 60 years) years) (*n*): ^m ^82/25/57

In comparison between 330 younger participants (< 60 years) and 697 older participants (≥ 60 years), the younger had higher prevalence of current depression (18% *vs* 9%), previous depression, anxiety, obesity (BMI ≥ 30 and ≥ 40 kg/m^2^), HbA1c > 64 mmol/mol, smoking (all *p* < 0.001); and antidepressant use and physical inactivity (both *p* = 0.003). The older participants had higher prevalence of pre-existing MI (*p* < 0.001) and stroke (*p* = 0.001).

Among the younger participants (< 60 years), 113 women compared to 217 men had higher prevalence of current depression (31% *vs* 12%), previous depression, and antidepressant use (all *p* < 0.001); anxiety (*p* = 0.001); obesity (BMI ≥ 30 and ≥ 40 kg/m^2^) (*p* = 0.023 and 0.017 respectively); and smoking (*p* = 0.029). The men had higher prevalence of HbA1c > 64 mmol/mol (> 8%) (*p* < 0.001). Among the older participants (≥ 60 years), 297 women compared to 400 men had higher prevalence of previous depression and antidepressant use (both *p* < 0.001); anxiety (*p* = 0.005); and obesity (BMI ≥ 30 and ≥ 40 kg/m^2^) (*p* = 0.048 and 0.008 respectively). The men had higher prevalence of pre-existing MI (*p* < 0.001) and stroke (*p* = 0.025). The prevalence of current depression did not differ between the older men and women (9%).

In Table 2, characteristics are compared between currently depressed and non-depressed participants, and for the following variables anxiety, antidepressant use, obesity, smoking, and physical inactivity the comparisons are also presented by sex.

**Table 2:** Comparisons between depressed and non-depressed presented for all, for 330 younger and 697 older patients with type 2 diabetes

		**Current depression (HADS-D ≥ 8) **^**a**^								
		**All patients (18–94 years)**			**Younger patients (18–59 years)**			**Older patients (60–94 years)**		
		**Yes**	**No**	***P*** ^**b**^	**Yes**	**No**	***P*** ^**b**^	**Yes**	**No**	***P*** ^**b**^
*n*		121	906		60	270		61	636	
Sex	Women	61 (50)	349 (38)	0.012	35 (58)	78 (29)	< 0.001	26 (43)	271 (43)	> 0.99
	Men	60 (50)	557 (62)		25 (42)	192 (71)		35 (57)	365 (57)	
Age		60 (50, 72)	66 (58, 72)	0.001 ^c^	36 (31, 39)	53 (46, 57)	0.003 ^c^	72 (66, 78)	70 (66, 75)	0.045 ^c^
Depression (previous) ^c^		61 (57)	130 (16)	< 0.001	36 (66)	47 (19)	< 0.001	25 (48)	83 (14)	< 0.001
Anxiety (HADS-A ≥ 8) ^d^	All	78 (64)	117 (13)	< 0.001	46 (77)	56 (21)	< 0.001	32 (52)	61 (10)	< 0.001
	Women	46 (75)	54 (16)	< 0.001	30 (86)	18 (23)	< 0.001	16 (62)	36 (13)	< 0.001
	Men	32 (53)	63 (11)	< 0.001	16 (64)	38 (20)	< 0.001	16 (46)	25 (7)	< 0.001
Antidepressants ^e^	All	31 (37)	61 (12)	< 0.001	22 (49)	21 (14)	< 0.001	9 (24)	40 (12)	0.043 ^f^
	Women	23 (49)	39 (19)	< 0.001	17 (59)	12 (24)	0.002	6 (33)	27 (18)	0.12 ^f^
	Men	8 (22)	22 (8)	0.010 ^f^	5 (31)	9 (9)	0.028 ^f^	3 (15)	13 (7)	0.18 ^f^
Obesity (BMI ≥ 30 kg/m^2^) ^g^	All	77 (66)	461 (53)	0.008	46 (82)	157 (61)	0.003	31 (52)	304 (50)	0.80
	Women	45 (75)	189 (57)	0.009	29 (85)	52 (68)	0.05	16 (62)	137 (54)	0.46
	Men	32 (57)	272 (51)	0.37	17 (77)	105 (58)	0.08	15 (44)	167 (47)	0.73
Obesity (severe) (BMI ≥ 40 kg/m^2^) ^g^	All	15 (13)	52 (6)	0.005	12 (21)	26 (10)	0.018	3 (5)	26 (4)	0.74 ^f^
	Women	12 (20)	27 (8)	0.005	10 (29)	10 (13)	0.038	2 (8)	17 (7)	0.85 ^f^
	Men	3 (5)	25 (5)	0.74 ^f^	2 (9)	16 (9)^e^	> 0.99 ^f^	1 (3)	9 (3)	0.60 ^f^
HbA1c > 64 mmol/mol (> 8%) ^h^		28 (24)	225 (25)	0.72	18 (31)	104 (39)	0.28	10 (16)	121 (19)	0.59
Smoking ^i^	All	23 (20)	104 (12)	0.012	15 (29)	46 (17)	0.10	8 (14)	58 (9)	0.29 ^f^
	Women	15 (25)	43 (13)	0.010	10 (30)	18 (24)	0.48 ^f^	5 (19)	25 (19)	0.16 ^f^
	Men	8 (14)	61 (11)	0.51 ^f^	5 (22)	28 (15)	0.37 ^f^	3 (9)	33 (9)	> 0.99^ f^
Physical inactivity ^j^	All	46 (43)	248 (29)	0.004	28 (52)	87 (34)	0.014	18 (33)	161 (27)	0.32
	Women	24 (45)	90 (27)	0.007	14 (45)	22 (29)	0.10	10 (46)	68 (27)	0.08 ^f^
	Men	22 (40)	158 (30)	0.15	14 (61)	65 (36)	0.023	8 (25)	93 (27)	> 0.99 ^f^
Myocardial infarction ^k^		13 (12)	82 (10)	0.52	3 (6)	10 (4)	0.71 ^f^	10 (18)	72 (12)	0.21 ^f^
Stroke ^l^		8 (7)	54 (6)	0.69 ^f^	3 (6)	5 (2)	0.16 ^f^	5 (9)	49 (8)	0.80 ^f^

Data are presented as *n* (%) or median (q_1_, q_3_)

^a^ Hospital Anxiety and Depression Scale-Depression subscale. ^b^ Pearson Chi-Square unless indicated. ^c^ Mann–Whitney U test. Missing values (all/ < 60 years/ ≥ 60 years) (*n*): ^c ^95/28/67. ^d^ Hospital Anxiety and Depression Scale-Anxiety subscale

Missing values (all/ < 60 years/ ≥ 60 years) (*n*): years): ^e ^453/138/315. ^f^ Fisher´s Exact Test. ^g ^Body Mass Index. Missing values (all/ < 60 years/ ≥ 60 years) (*n*): ^g^ 45/16/29. ^h^ Hemoglobin A1c. Missing values (all/ < 60 years/ ≥ 60 years) (*n*): ^h^10/2/8; ^i^ 23/9/14; ^j^ 68/20/48; ^k^ 86/28/58; ^l^ 82/25/57

In all participants, the 121 currently depressed compared to 906 non-depressed participants were younger (*p* = 0.001) and more often women (*p* = 0.012); and they had higher prevalence of previous depression, anxiety, antidepressant use (all *p* < 0.001); physical inactivity (*p* = 0.004); obesity (BMI ≥ 30 and ≥ 40 kg/m^2^) (*p* = 0.008 and 0.005 respectively); and smoking (*p* = 0.012).

Among the younger participants (< 60 years), the 60 currently depressed compared to 270 non-depressed participants were more often women and had higher prevalence of previous depression, anxiety, and antidepressant use (all *p* < 0.001); obesity (BMI ≥ 30 and ≥ 40 kg/m^2^) (*p* = 0.003 and 0.018 respectively); and physical inactivity (*p* = 0.014). The highest prevalence of severe obesity (BMI ≥ 40 kg/m^2^) (29%) was found in the young depressed women. Among the older participants (≥ 60 years), the 61 currently depressed compared to 636 non-depressed participants were older (*p* = 0.045) and had higher prevalence of previous depression and anxiety (both *p* < 0.001); and antidepressant use (*p* = 0.035).

In Table 3, associations with current depression are presented.

**Table 3:** Associations with current depression presented for all, younger and older, women and men, with type 2 diabetes

	**Current depression (HADS-D ≥ 8)** ^a^									
	**All patients** **(18–94 years)**		**Younger women **^**b**^ **(18–59 years)**		**Younger men **^**c**^ **(18–59 years)**		**Older women **^**d**^ **(60–94 years)**		**Older men **^**e**^ **(60–94 years)**	
	**OR (95% CI)**	***P***^** f**^	**OR (95% CI)**	***P***^** f**^	**OR (95% CI)**	***P***^** f**^	**OR (95% CI)**	***P***^** f**^	**OR (95% CI)**	***P***^** f**^
Age (per year)	0.97 (0.96–0.98)	0.001	0.92 (0.88–0.97)	0.001	0.99 (0.94–1.04)	0.65	1.01 (0.96–1.07)	0.68	1.08 (1.02–1.14)	0.004
Sex (women)	1.6 (1.1–2.4)	0.013	-	-	-	-	-	-	-	-
Depression (previous)^ g^	7.1 (4.6–10.9)	< 0.001	12.1 (4.3–33.9)	< 0.001	4.0 (1.6–10.1)	0.003	9.3 (3.4–25.5)	< 0.001	4.6 (2.0–10.4)	< 0.001
Anxiety (HADS-D ≥ 8)^ h^	12.2 (8.0–18.6)	< 0.001	20.0 (6.8–59.1)	< 0.001	7.2 (3.0–17.6)	< 0.001	10.4 (4.4–24.8)	< 0.001	11.5 (5.3–25.0)	< 0.001
Antidepressants ^i^	4.2 (2.5–7.1)	< 0.001	4.5 (1.7–12.0)	0.003	4.4 (1.3–15.7)	0.020	2.3 (0.8–6.7)	0.13	2.4 (0.6–9.4)	0.20
BMI (per unit) (kg/m^2^)^ j^	1.07 (1.04–1.11)	< 0.001	1.08 (1.01–1.14)	0.020	1.04 (0.97–1.12)	0.27	1.04 (0.98–1.12)	0.22	1.02 (0.94–1.10)	0.63
Obesity (BMI ≥ 30 kg/m^2^) ^j^	1.7 (1.2–2.6)	0.008	2.8 (1.0–8.1)	0.058	2.5 (0.9–7.0)	0.090	1.4 (0.6–3.1)	0.46	0.9 (0.4–1.8)	0.73
Obesity (severe) (BMI ≥ 40 kg/m^2^) ^j^	2.3 (1.3–4.3)	0.007	2.8 (1.0–7.5)	0.043	1.0 (0.2–4.8)	0.97	1.2 (0.3–5.3)	0.85	1.2 (0.1–9.5)	0.89
HbA1c (per mmol/mol)^ k^	1.00 (0.99–1.01)	0.92	1.01 (0.99–1.04)	0.18	1.00 (0.98–1.01)	0.75	0.98 (0.95–1.01)	0.23	1.00 (0.98–1.02)	0.97
Smoking^ l^	1.9 (1.1–3.1)	0.013	1.4 (0.6–3.5)	0.47	1.6 (0.5–4.7)	0.39	2.3 (0.8–6.6)	0.12	1.0 (0.3–3.4)	0.98
Physical inactivity ^m^	1.8 (1.2–2.7)	0.005	2.1 (0.9–4.9)	0.10	2.7 (1.1–6.7)	0.027	2.3 (0.9–5.5)	0.066	0.9 (0.4–2.0)	0.78

^a^ Hospital Anxiety and Depression Scale-Depression subscale. *N* = 1027 for all, n for 4 subgroups: ^b ^113/^c ^217/^d ^297/^e ^400 unless indicated

^f^ Logistic regression analyses (univariate). Missing values: ^g ^(^b ^9/^c ^19/^d ^32/^e ^35). ^h^ HADS-A Hospital Anxiety and Depression Scale-Anxiety subscale

Missing values: ^i^ (^a^34/^b^104/^c^128/^d^187). ^j^ Body Mass Index. Missing values: ^j ^(^a^2/^b^14/^c^17/^d^12). ^k^ Hemoglobin A1c. Missing values: ^k ^(^a^2/^b^0/^c^3/^d^5); ^l ^(^a^4/^b^5/^c^5/^d^9); ^m ^(^a^5/^b^15/^c^20/^d^28)

In all participants, age (per year) (inversely) (OR 0.97 (CI 95% (0.96–0.98)), women (OR 1.6 (CI 95% 1.1–2.4)), anxiety (OR 12.2 (CI 95% 8.0–18.6)), previous depression (OR 7.1 (CI 95% 4.6–10.9)), antidepressant use (OR 4.2 (CI 95% 2.5–7.1)), obesity (BMI ≥ 30 and ≥ 40 kg/m^2^) (OR 1.7 (CI 95% 1.04–1.11) and OR 2.3 (CI 95% 1.3–4.3)respectively), smoking (OR 1.9 (CI 95% 1.1–3.1)), and physical inactivity (OR 1.8 (CI 95% 1.2–2.7)), were associated with depression (all *p* ≤ 0.013).

In younger women (< 60 years), age (per year) (inversely) (OR 0.92 (CI 95% 0.88–0.97)), anxiety (OR 20.0 (CI 95% 6.8–59.1)), previous depression (OR 12.1 (CI 95% 4.3–33.9)), antidepressant use (OR 4.5 (CI 95% 1.7–12.0)), obesity (BMI ≥ 30 kg/m^2^ (OR 2.8 (CI 95% 1.0–8.1)) and (BMI ≥ 40 kg/m^2^ OR 2.8 (CI 95% 1.0–7.5)), were associated with depression (all *p* ≤ 0.043). In younger men (< 60 years), anxiety (OR 7.2 (CI 95% 3.0–17.6)), antidepressant use (OR 4.4 (CI 95% 1.3–15.7)), previous depression (OR 4.0 (CI 95% 1.6–10.1)), and physical inactivity (OR 2.7 (CI 95% 1.1–6.7)), were associated with depression (all *p* ≤ 0.027).

In both older women and men (≥ 60 years), only anxiety (OR 10.4 (CI 95% 4.4–24.8) and OR 11.5 (CI 95% 5.3–25.0) respectively) and previous depression (OR 9.3 (95% 3.4–25.5) and OR 4.6 (CI 95% 2.0–10.4) respectively) were associated with depression (all *p* ≤ 0.001)*.*

## Discussion

In this Swedish multicentre study of depression in 1027 participants with newly diagnosed T2D, younger age, women, previous depression, anxiety, antidepressant use, obesity (both BMI ≥ 30 and ≥ 40 kg/m^2^), smoking, and physical inactivity, were associated with current depression. There were distinct differences between younger and older participants. Younger participants (< 60 years) had significantly higher prevalence of current and previous depression, anxiety, antidepressant use, obesity (both BMI ≥ 30 and ≥ 40 kg/m^2^), high HbA1c (> 64 mmol/mol), smoking, and physical inactivity, than older participants. There were also sex differences with higher prevalence of both current and previous depression, anxiety, antidepressant use, obesity (both BMI ≥ 30 and ≥ 40 kg/m^2^), and smoking in the younger women, but higher prevalence of HbA1c > 64 mmol/mol (> 8%) in the younger men. In the younger women, previous depression, anxiety, antidepressant use, and severe obesity were associated with current depression. In the younger men, physical inactivity in addition to previous depression, anxiety, and antidepressant use, were associated with depression. In the older participants, the prevalence of current depression did not differ between women and men, but the older women had significantly higher prevalence of previous depression, antidepressant use, and obesity (both BMI ≥ 30 and ≥ 40 kg/m^2^) than the older men.

To our knowledge, the prevalence of depression at the time of diagnosis of T2D has not previously been explored in Sweden. According to previous research, depression is a risk factor for incident T2D [2, 3] and for cardiovascular and all-cause mortality [1]. Depression as a risk factor for incident T2D seems to be as important as smoking and physical inactivity [3]. The presence of anxiety in depressed patients seems to further increase the risk of incident T2D [4]. In this study the association between depression and anxiety was very robust in all subgroups. The prevalence of current depression (12%) in all participants was the same as the prevalence of depression in the Swedish population study (11.7%), where depression was defined as in the present study (HADS-D ≥ 8) [8]. However, the prevalence of current depression in the younger women (< 60 years) (31%) with newly diagnosed T2D was 2.6 times higher than for younger women in the Swedish population study (Swedish women: 18–84 years 11.2%, 18–64 years 11.9%, ≥ 64 years 9,4%) [8], while the younger men with newly diagnosed T2D had the same prevalence of depression (12%) as younger men in the Swedish population study (Swedish men: 18–84 years 12.3%, 18–64 years 12.2%, ≥ 64 years 13.2%) [8]. The prevalence of current depression in the older participants (≥ 60 years) (both men and women 9%) was approximately the same as for older women in the Swedish population study, but lower for the older men with T2D than in the Swedish population [8]. On the other hand, a large proportion of the older participants, particularly the women, reported being depressed previously. In another Swedish study, the prevalence of depression (HADS-D ≥ 8), was 13% in patients hospitalized due to coronary heart disease and 4% in healthy controls [18]. Thus, the younger women with newly diagnosed T2D had 2.4 times higher prevalence of depression (31%) than patients with coronary heart disease (13%), and 7.8 times higher prevalence of depression than the healthy controls (4%). In a Swedish study of depression (HADS-D ≥ 8) in patients with T1D aged 18–59 years, the prevalence of depression in the women was 11% and 10% in the men [24], rendering a depression prevalence 2.8 times higher in the younger women and 1.2 higher in the younger men with newly diagnosed T2D in this study.

The prevalence of antidepressant use in our study was higher for younger women (4 times higher), older women (3.3 times higher), younger men (2.5 times higher), and for older men (1.4 times higher) than in the Swedish population (Swedish women/men: 18–84 years 9.8%/men 5.3%), 18–64 years 9.0%/4.8%, ≥ 64 years 10.1%/5.6%) [8]. Younger T2D patients had also higher prevalence of antidepressant use than T1D patients aged 18–59 years (8%) [24, 25].

Obesity is another major contributor to both incident T2D [9, 10, 14, 26] and to CVD and mortality [26]. The total prevalence of obesity (BMI ≥ 30 kg/m^2^) in the Swedish general population during the period 2016–2017 was 16.6% (women 14.5%, men 18.1%) [21]. In our study the prevalence of obesity (BMI ≥ 30 kg/m^2^) was higher both in depressed and non-depressed participants compared to the Swedish general population. The highest prevalence of obesity was demonstrated in the depressed younger women (85%), 5.9 times higher than for women in the Swedish population (14.5%) [21]. The demonstrated association between obesity and depression in this study of T2D differs from findings in patients with T1D, where no association between depression and obesity was found [27].

Furthermore, smoking is a major risk factor for both incident T2D [15], and for CVD and mortality (27). In 2016, the prevalence of smoking was 9% in the Swedish population [28], which can be compared to 13% in all participants, and 20% in the depressed and 12% in the non-depressed participants, with the highest prevalence in the younger depressed participants (29%).

Physical inactivity is another important contributor to both incident T2D [9, 10] and cardiovascular and all-cause mortality [29]. The prevalence of physical inactivity (less than 30 min of moderate physical activity once a week) was high in the participants (31%) compared to Swedish T1D patients (13%) [24]. Physical inactivity was particularly common in the young depressed participants in our study (52%). We have no data from the Swedish general population of the prevalence of physical inactivity defined as in our study.

As T2D usually has a long pre-detection period of 3–7 years [10], and as we do not know for how long the participants in our study had experienced depressive symptoms or used antidepressants, we cannot determine whether depression preceded or succeeded the onset of T2D. Due to the cross-sectional design, no causality can be drawn from our results.

Clinically, as well as in further research, it is probably important to detect depression and explore potential underlying conditions in patients with newly diagnosed T2D, particularly in young persons, in order to provide optimal treatment. Underlying conditions could be either somatic, psychological and/or social. Increased cortisol secretion has been demonstrated in both depressed and obese patients [6, 7, 30, 31], and cortisol secreting tumours may induce obesity and T2D [32]. Weight stigma and discrimination are linked to both depression and to binge eating disorder [33], which has a high life-time risk for developing T2D [34]. Post-traumatic stress disorder (PTSD) has also been linked to depression [35], obesity [36], and T2D [37]. Attention deficit/hyperactivity disorder (ADHD) is another disorder previously associated with depression [38], obesity [39], and T2D [40]. Additionally, in further research, exploration of shared endocrine and inflammatory disturbances in patients with depression, obesity and T2D would be of interest. We intend to explore the food habits of these participants in a separate article.

One strength of our study is the multicentre recruitment from a large number of health care units in both urban and rural areas in two separate Swedish regions during two years. The clinical diagnoses of T2D were confirmed as all included participants had remaining insulin secretion without immunological signs of autoimmunity [16]. By including questions about previous depression and the use of antidepressants, we could show that depression was not just a reaction to the T2D diagnosis. Since previous research indicated increasing prevalence of severe obesity [22], two levels of obesity were reported. Relevant variables were included as depression previously has been linked to weight gain and obesity, particularly in atypical depression [6, 7], incident T2D [2–4], high HbA1c levels [24], physical inactivity [41], smoking [42], coronary heart disease [1, 17], stroke [43], and all-cause mortality [1]. Age is relevant as incident T2D increases with age [9], but younger-onset T2D is particularly harmful with increased mortality [13]. The increased prevalence of depression, obesity, smoking, and physical inactivity, may all be explanatory factors to the increased mortality previously demonstrated in younger-onset T2D [13]. Age divided into 7 age-groups made it possible to study the details of the distribution of depression and obesity, and also facilitated determining a suitable cut-off for further analyses. The prevalence of depression declined until the age-group 60–69 years, which indicated that the age of 60 years was a suitable cut-off level for the age analysis.

A major limitation to our study was the high number of non-respondents to the question “Do you take antidepressant medication?” Other limitations were that there was no information regarding type of antidepressants and duration of use. Also, current depression was not confirmed by a structured interview. Yet, HADS-D has shown high validity for assessing depressive symptoms both at an individual and a collective level [20]. HADS does not include symptoms that could be signs of a somatic disease accompanied by weight changes [19], and is extensively used in research [8, 17–20, 24, 25]. Patients with inadequate knowledge of Swedish were excluded, as we could not guarantee the quality of the translation of HADS into other languages. This exclusion criteria probably contributed to underrepresentation of immigrants. According to recent research, first generation immigrants constitute about 21% of newly diagnosed T2D in Sweden [44], compared to 12% in this study. This is important as nationwide Swedish studies have shown increased risk of depression in immigrants [45]. Though the total number of included patients was quite high, it is still a limitation that just 29% of patients with newly diagnosed clinical T2D were included. However, the percentage might have been higher if only serologically verified T2D had been included in the total number. The reasons for non-participation were not registered systematically, but several health care units have reported they lacked time to include all new patients due to staff shortage. Other reasons for non-participation were that patients did not wish to participate, their T2D diagnoses were not serologically verified, or their HADS questionnaires were not adequately completed.

## Conclusions

The younger women had the highest prevalence of depression, anxiety, and severe obesity. The prevalence of depression in young women and antidepressant use in all patients were higher than in the Swedish general population. Three risk factors for CVD, obesity, smoking, and physical inactivity, were associated with depression.

## Data Availability

The data set analyzed during the current study is not available publicly as individual privacy could be compromised, and we have no permission from the Regional Ethical Board to share the research data publicly. The data set is stored at the department for Research and Development, Region Kronoberg, Växjö, Sweden, and is available from the corresponding author on reasonable request.

## Abbreviations

BMI: Body Mass Index
CI: Confidence Interval
EHRs: Electronic health records
ELISA: Enzyme linked immunosorbent assay
GAD: Glutamic acid decarboxylase
HbA1c: Hemoglobin A1c
HADS-A: Hospital Anxiety and Depression Scale-anxiety subscale
HADS-D: Hospital Anxiety and Depression Scale-depression subscale
MI: Myocardial infarction
OGTT: Oral glucose tolerance test
OR: Odds ratio
S-NDR: Swedish National Diabetes Register
T1D: Type 1 diabetes
T2D: Type 2 diabetes
